# Second language proficiency modulates conflict-monitoring in an oculomotor Stroop task: evidence from Hindi-English bilinguals

**DOI:** 10.3389/fpsyg.2013.00322

**Published:** 2013-06-12

**Authors:** Niharika Singh, Ramesh K. Mishra

**Affiliations:** Centre of Behavioural and Cognitive Sciences, University of AllahabadAllahabad, India

**Keywords:** bilingualism, conflict-monitoring, language proficiency, saccades, Stroop task

## Abstract

Many studies have confirmed the presence of a bilingual advantage which is manifested as enhanced cognitive and attention control. However, very few studies have investigated the role of second language proficiency on the modulation of conflict-monitoring in bilinguals. We investigated this by comparing high and low proficient Hindi-English bilinguals on a modified saccadic arrow Stroop task under different monitoring conditions, and tested the predictions of the bilingual executive control advantage proposal. The task of the participants was to make an eye movement toward the color patch in the same color as the central arrow, ignoring the patch to which the arrow was pointing. High-proficient bilinguals had overall faster saccade latency on all types of trials as compared to the low proficient bilinguals. The overall saccadic latency for high proficiency bilinguals was similarly affected by the different types of monitoring conditions, whereas conflict resolution advantage was found only for high monitoring demanding condition. The results support a conflict-monitoring account in a novel oculomotor task and also suggest that language proficiency could modulate executive control in bilinguals.

## Introduction

Bilingualism is a widespread socio-cultural phenomenon in the world today. Most people learn a second language and become bilingual for different professional, social as well as cultural reasons. Several studies have found a bilingual cognitive control advantage on non-linguistic tasks (Bialystok and Martin, 2004; Bialystok et al., 2005, 2006; Carlson and Meltzoff, 2008; Colzato et al., 2008; Costa et al., 2008, 2009; Martin-Rhee and Bialystok, 2008; Bialystok and Feng, 2009; Bialystok and Viswanathan, 2009; Kovács and Mehler, 2009; Ye and Zhou, 2009; Bialystok, 2010; Festman et al., 2010; Hernández et al., 2010). Even bilingual infants have been shown to demonstrate superior cognitive control skills than monolingual infants (Kovács and Mehler, 2009). However, the exact locus of the bilingual cognitive control advantage is still not understood in a variety of situations. For example, it is still not clear if bilingualism enhances inhibitory control mechanisms in particular or the general executive control systems that allow them to be faster in situations even when there is no apparent conflict. There have been recent proposals (Bialystok, 2010; Hilchey and Klein, 2011) that suggest bilinguals in general have enhanced attentional control system and not a specific response inhibition mechanism. In this research we particularly explore the “bilingual executive control advantage” theory to see if language proficiency modulates conflict-monitoring in an oculomotor Stroop task when monitoring demands are manipulated.

### The bilingual executive control advantage

The central assumption behind bilingual's cognitive control advantage on non-linguistic tasks stems from the fact that bilinguals need to manage their two languages efficiently and select the right lexicon during language production. The inhibitory control model of Green (1998) predicts superior conflict resolution between competing language nodes in bilinguals. On this account, on a non-linguistic conflict task like the Stroop task, bilinguals should show some advantage while processing the incongruent trials. However, a close inspection of the performance on different trials in different conflict tasks shows that bilinguals are not only faster on incongruent trials but also on congruent and neutral trials, where there is no conflict (Bialystok et al., 2006; Costa et al., 2008, 2009; Martin-Rhee and Bialystok, 2008). In some other studies an overall RT advantage also accompanies reduced conflict costs and facilitation (Hernández et al., 2010 experiment 1; Singh and Mishra, 2012). Luk et al. (2010) administered a spatial Flanker Task and a go no-go task to bilinguals and monolinguals. The behavioral RTs showed no differences between the bilinguals and monolinguals. However, the neuroimaging results showed that bilinguals are better in interference suppression but not on response inhibition. This pattern of results shows that the bilingual advantage is specific to some type of cognitive control. The result showed that brain networks for bilinguals and monolinguals differed for the interference suppression task but not for the response inhibition task.

Bilingual's superior performance on congruent and neutral trials in a conflict task is problematic for an inhibitory control account. Hilchey and Klein (2011) in an influential meta-analysis of several bilingual cognitive advantage studies observed that most studies have found a global RT advantage for bilinguals but not many have found statistically significant reduced conflict or facilitation effects. This led Hilchey and Klein (2011) to propose that bilinguals as such may not be exercising any inhibitory control but may have enhanced attentional mechanisms and goal-maintenance abilities. Hilchey and Klein's Bilingual Executive Process Advantage claims that, bilinguals will demonstrate domain general executive control advantage on all kinds of trials including trials that have conflict. This advantage generally shows up in faster RTs on tasks that require some form of interference control and may or may not have any conflict. Most crucially, the executive control advantage theory emphasizes on bilingual's excellent goal directed attention control. This does not necessarily refer to an ability to exert reactive forms of inhibitory control on tasks that have conflict. A superior executive control system also allows top-down attention control on tasks that require goal maintenance, monitoring as well as interference suppression. Bilinguals' excellent performance on congruent trials could stem from their ability to take facilitative cues from the context (Bialystok, 2010). Now, the question is: under what conditions can we observe the Bilingual Executive Control Advantage?

It is important to note that the overall RT advantage seems to emerge when the tasks are cognitively demanding. One way to make the task more demanding is to manipulate the monitoring required for the task. Monitoring has been considered an important part of the executive control system (Posner, 1994). Costa et al. (2009) had examined bilinguals and monolinguals on the Flanker Task in high and low monitoring conditions. The low monitoring condition had higher proportion of either congruent or incongruent trials and the high monitoring condition had congruent and incongruent trials in equal proportion. The rationale was that low monitoring condition will not demand higher alertness and monitoring, and therefore will not tax the bilingual executive system. The results showed an overall RT advantage for bilinguals only for the high monitoring condition but not for the low monitoring condition. In this study, the bilinguals showed reduced conflict cost only in the block that had 75% congruent condition. Costa et al. (2009) concluded that bilingual overall RT advantage is seen only when the monitoring context had high uncertainty and was demanding. Further, it has also been suggested that the bilinguals may show advantage for congruent trials only for mixed blocks of trials, since this calls for constant monitoring and goal-maintenance (Bialystok et al., 2006; Costa et al., 2008; Bialystok, 2010). An earlier study by Bialystok and Martin (2004) found global RT advantage in a task that did not have any explicit conflict but participants operated under higher cognitive load.

There is other evidence which suggests that bilingualism could not just strengthen an inhibitory control system but may influence the attention system in general. Emmorey et al. (2009) had compared bimodal and unimodal bilinguals with monolinguals on a modified Flanker's task that had go no-go blocks and conflict trials. There was no group-difference for the response inhibition conditions, whereas unimodal bilinguals were faster on both incongruent and congruent trials. This led the authors to conclude that bilingual executive control advantage strengthens the interference suppression system so that they can better manage the conflict, while there is no evidence of a superior response inhibition mechanism. These data suggest that bilingual advantage is more prominent where some complex decision-making is involved that is reached through efficient control of interference. The general executive control advantage theory of Hilchey and Klein (2011) can accommodate these observations since these data do not point toward an exclusive inhibitory control mechanism and show general RT advantage with, or without, specific conflict, or facilitation, advantage.

Some other proposals also point toward bilinguals having a superior attentional-control system, which is part of the general executive control system. Colzato et al. (2008) compared bilinguals and monolinguals on the stop signal, the IOR, and the attentional blink (AB) task. Interestingly, they could not find any difference between the two groups on the stop signal task, supposedly a task that measures inhibitory control, but bilinguals showed a high AB effect. It was interpreted that a higher AB effect suggests that bilinguals are better at keeping goal-directed information, and at suppressing the unwanted stimuli from further processing. It has been also shown that bilinguals are better in avoiding distraction that may come from maintaining an item in working memory during a visual search task (Hernández et al., 2012) and attention control during dichotic listening (Soveri et al., 2011). These examples of top down attention control and goal-maintenance indirectly lend support to the general claims of the executive control account in showing that bilingualism enhances the ability to suppress interference increases acting in a focused manner.

In summary, the bilingual executive control account which predicts overall RTs for all types of trials can account for a range of findings. However, most such findings have come from studies where bilinguals and monolinguals have been compared. In the present study, we wanted to further extend this proposal in testing if bilingual language fluency could modulate the overall RT advantage in the oculomotor domain. Below, we review studies where language proficiency has been an issue in the studies of executive control system.

### Bilingual language proficiency and executive control

Why should bilingual fluency enhance executive control on non-linguistic conflict tasks? The rationale behind linking higher proficiency to enhanced executive control lies in the observation that highly fluent bilinguals have constant experience of handling cross-linguistic influences from two lexicons. Bilinguals need to manage these cross-linguistic influences, since they activate two lexicons in parallel in a language non-selective manner. However, this level of activation varies with proficiency and therefore this should modulate the demand for the executive control system to intervene. Blumenfeld and Marian (2007) used the visual world eye tracking paradigm with German-English and English-German bilinguals to examine the influence of language proficiency on parallel lexicon activation. The results showed that only highly fluent German-English bilinguals activated German while processing English-specific targets. Similarly, others have shown that higher language proficiency produces stronger parallel activation of lexicons in bilinguals (Jared and Kroll, 2001; Van Hell and Dijkstra, 2002). ERP studies with naming have shown that language proficiency of bilinguals affect the time course of parallel activation of the non-target lexicon (Guo and Peng, 2006). Recent studies have shown that highly fluent bilinguals activate translation equivalents of the non-target languages unconsciously (Thierry and Wu, 2010; Guo et al., 2012; Sunderman and Priya, 2012). Given this evidence it is logical to assume that higher language proficiency will lead to higher executive control.

Tao et al. (2011) looked at how age of L2 acquisition influenced executive control in early and late bilinguals. Their results on the lateralized attention network task showed that low proficient bilinguals were better at the monitoring processes while the late and balanced bilinguals had greater conflict resolution advantage. In sum, it appears that age of acquisition and current proficient both have an effect on the executive control systems in bilinguals. It is not necessarily true that bilinguals who acquired their L2 later are non-balanced and vice-versa. Bilinguals who are more balanced may require a different kind of inhibitory control mechanism, since they may activate unnecessary lexicons to different degrees than bilinguals who are less balanced. Therefore, it is important to explain bilingual cognitive advantage data on non-linguistic stimuli from these perspectives. In another relevant study, Festman and Münte (2012) studied late but fluent bilinguals who differed in their switching behavior. Language switching has been known to be linked to bilingual's proficiency. Festman and Münte (2012) observed that late high-proficient bilinguals who switched less had higher conflict resolution and superior executive control. However, it looks like for highly fluent bilinguals their abilities to switch between linguistic items and non-linguistic items and the amount of control they need may be different. Calabria et al. (2011) compared highly proficient bilinguals on both linguistic and non-linguistic switch tasks. These bilinguals demonstrated symmetrical switch costs for the linguistic task while it was not so for the non-linguistic task. Based on these results Calabria et al. (2011) argued that bilinguals' language control system could be different from their overall executive control system, or may even be a subsidiary.

Bialystok et al. (2006) investigated presence of bilingual advantage on executive function component related to monitoring and switching in High-proficient bilinguals, unbalanced bilinguals and the control group of monolinguals. Participants had to classify objects in the visual modality while simultaneously processing auditory information. Interestingly, the scores for the unbalanced bilinguals lied midway between bilinguals and monolinguals group, but no reliable and significant difference was observed for the unbalanced bilinguals. The study showed fluent bilinguals being better at monitoring. Luk et al. (2011) compared late bilinguals, early bilinguals and monolinguals on a Flanker Task. The results showed early bilinguals demonstrating smaller interference costs for the incongruent trials while late bilinguals were similar to monolinguals. These studies indicate that bilinguals' language experience and fluency could have some influence on the executive control system.

Singh and Mishra (2012) compared two groups of Hindi-English bilinguals who differed in their L2 proficiency on an oculomotor version of the Stroop task (Hodgson et al., 2009). Participants were asked to make an eye movement toward the color patch that was similar to the color in which the centrally presented color word was written resisting interference from the meaning of the word. The results showed that bilinguals with superior L2 proficiency had an overall speed advantage and higher conflict resolution compared to low proficient bilinguals. Interestingly a previous study by Bialystok et al. (2006) did not find any bilingual advantage for an anti-saccade task where eye movements were recorded while an advantage was seen when in the same task participants had to make a manual response. In another study, Mishra et al. (2012) used the classic Posner's cuing paradigm (Posner, 1980) to study if high-proficient Hindi-English bilinguals could disengage their attention from an uninformative peripheral cue and thus would indicate the presence of better endogenous attention disengagement. The results revealed that high-proficient bilinguals showed an early appearance of IOR compared to low proficient bilinguals, suggesting their ability to disengage their attention from a task-irrelevant cue. These studies provide evidence for the fact that those bilinguals who have achieved a superior fluency in their language skills demonstrate better attentional control on tasks that demand either conflict resolution or disengagement of their attention. Others have shown that language fluency could influence goal maintenance and conflict resolution (Coderre et al., 2012; Tse and Altarriba, 2012). However, none of the previous studies have examined if language proficiency affects the overall RT advantage in a conflict task.

### The current study

In this study we examined how language proficiency might modulate monitoring during a conflict resolution task in Hindi-English bilinguals who differed exclusively in their L2 proficiency. The oculomotor Stroop task that we used (see Singh and Mishra, 2012) requires participants to make an eye movement toward a color patch that is similar to the arrow's color while resisting interference generated from the arrow's direction. In essence the task requires resolving the conflict between these two opposite responses. We also manipulated the trial compositions of congruent and incongruent trials to test the predictions of the monitoring account of bilingual executive control in participants who differed on their current language proficiency. If Costa et al. (2009) findings are to be replicated in this oculomotor version of the Arrow Stroop task, we should expect overall RT advantages for the highly fluent group in the block where congruent and incongruent trials are of equal number. As for the reduced conflict effect, the question remains open since it has been shown to depend on particular contexts. Further, we expected the highly fluent group to commit fewer errors.

Although Singh and Mishra (2012) had examined an oculomotor version of the Stroop task with bilinguals with two different language proficiencies, the study was not an explicit test of the conflict-monitoring account. Secondly, Singh and Mishra (2012) had used written words and it remains a possibility that linguistic processing of words might have played some role in the adjustment of control processes. In this study we used colored arrows to reflexively manipulate attention and create conflict which we assumed would be a proper non-linguistic task (Hilchey and Klein, 2011 recommend this task as a suitable task to measure conflict-monitoring). Recent literature suggests that certain biological cues, such as eye gaze, and highly learned social symbols such as arrows or spatial words (right, left etc.), can lead to a shift of attention reflexively even when these cues are task irrelevant (Friesen and Kingstone, 1998; Driver et al., 1999; Langton and Bruce, 1999; Langton et al., 2000; Kunde et al., 2011). Centrally presented arrow cues produce reflexive shifts of attention in the direction of the arrow (Ristic et al., 2002; Tipples, 2002). We expected that highly fluent bilinguals will override the reflexive shifts of attention induced by central arrow cues using their top-down attention control strategies (Friesen et al., 2004).

## Method

### Participants

Fifty-six Hindi–English bilinguals participated in the experiment. All the participants were native speakers of Hindi (L1) and acquired English as a second language formally through instruction at school. Based on their proficiency ratings and responses made to the language proficiency questionnaire the participants were assigned to low (Mean age = 19.8, *SD* = 2.2) and high (Mean age = 21.9, *SD* = 2.6) proficiency bilingual groups. The proficiency of participants for both L1 and L2 was assessed by a self-devised language proficiency questionnaire, which required the participants to provide information related to age of acquisition of L2, daily exposure to both the languages (1 = rarely exposed, 2 = sometimes exposed, 3 = most often exposed), daily usage of languages for work-related activities and their daily percentage exposure to both the languages (see Table 1). The participant also rated their L1 and L2 language proficiencies for speaking, understanding, reading and writing ability on a Likert scale of 5 (where 1 represented “*poor*” and 5 represented “*excellent*”) (See Table 2).

**Table 1:** **Demographic details and mean non-verbal IQ of high-proficient bilinguals (HPB) and low proficient bilinguals (LBP)**.

	**HPB**	**LPB**
Mean formal age of L1 acquisition (years)	3.5 (0.65)	3.8 (0.77)
Mean formal age of L2 acquisition (years)	3.5 (0.82)	4.2 (0.75)
Exposure to L1^**^	3 (0.0)	2.8 (0.35)
Exposure to L2^**^	2.8 (0.35)	2.0 (0.47)
Hours of work related activity in L1^**^	2.8 (2.0)	4.3 (2.6)
Hours of work related activity in L2^**^	4.7 (1.9)	2.5 (2.3)
Mean score in L1 comprehension (out of 5)	4.7 (0.51)	4.3 (1.1)
Mean score in L2 comprehension (out of 5)^**^	4.3 (0.77)	2.8 (1.2)
Non-verbal IQ	54.3 (3.3)	53.7 (2.5)
Socio-economic status	2.3 (0.66)	2.17 (0.47)

Standard deviations are given in parentheses.

Note: L1, Hindi; L2, English; Non-verbal IQ: Ravens Progressive Matrices, out of 60. See text for explanation.

^*^p < 0.05;

** p <0.01.

**Table 2:** **Self-ratings for reading, writing, speaking, and comprehension in L1 and L2**.

	**Speaking**		**Listening**		**Reading**		**Writing**	
	**L1**	L2^**^	**L1**	L2^**^	**L1**	L2^**^	**L1**	L2^**^
HPB	4.7 (0.46)	3.7 (0.87)	4.7 (0.44)	4.3 (0.72)	4.5 (0.57)	4.4 (0.57)	4.3 (0.68)	4.1 (0.62)
LPB	4.6 (0.47)	2.8 (0.80)	4.7 (0.41)	3.1 (0.91)	4.7 (0.44)	3.4 (0.93)	4.4 (0.63)	3.1 (0.91)

Standard deviations are given in parentheses.

Note: L1, Hindi; L2, English; high and low proficiency based on L2 proficiency.

^*^p < 0.05;

** p < 0.01.

Apart from this, participants' proficiency was also established by their scores obtained on a reading comprehension in both the languages (See Table 1). An effort was taken that the two groups were well matched on the factors like SES and non-verbal IQ (Raven's progressive matrices), which are known to modulate cognitive control (Emmorey et al., 2009). Socioeconomic status of the participants was determined by asking participants to indicate, on a 3 point scale, to which socio-economic group they belonged (1 for *lower middle class*, 2 for middle class and 3 for *high middle class*). The *t*-tests conducted to compare the two groups on the non-verbal IQ test and SES revealed no significant difference between SES for the two groups (see Table 1).

It is important to briefly discuss the socio-linguistic aspects of language use of the participants. These participants study at a University where language of instruction is English. They use Hindi socially and often at home. However, the high and the low proficient participants are different in terms of their use of English in and outside work place. All the participants spoke the standard variety of Hindi. None of the participants knew another language and could not be considered multi-lingual. Very frequently these bilinguals code mix while using language.

### Stimuli

The stimuli consisted of a display containing four squares in four different colors (blue, black, green, and red) along with a central arrow presented at the center of the screen. The four squares were presented at four different locations i.e., up, down, left, and right and each cultured square subtended 1.6° of arc at an eccentricity of 7.3° from the center of a screen. These color patches were fixed at their locations for all trials. There were three experiment trials (congruent, incongruent and neutral). The arrow extending 0.96 × 0.23 cm at the center, could point at any of the four squares in any of the four locations, however, the combination of color of and direction central arrow decided the congruency of the trials. In the congruent condition the color and direction of arrow pointed at the same square (e.g., red color arrow pointing toward red square) whereas in the incongruent trials they corresponded to different squares (e.g., red cultured arrow pointing toward green square). In the neutral trials, the central arrow was replaced by cultured equi-sized (vertical or horizontal) lines which could match with any of the four square patch color. However, for all the types of trial the participants were instructed to look at the square that matched the color of the arrow while ignoring the square at which it was pointing.

Apart from this, three different monitoring conditions were created by manipulating the congruent proportion. There were three conditions: (1) 50% congruence proportion condition in which congruent (90) and incongruent trials (90) were equal in number, (2) 80% congruence proportion condition in which congruent (144) trials were more than incongruent trials (36), and (3) 20% congruence proportion condition consisted of more incongruent (144) trials than congruent trials(36). For all the conditions the number of neutral trials (90) was fixed. Each condition consisted of 270 trials in total and was presented as separate block.

### Apparatus and procedure

Eye movements were monitored using IView X high-speed eye tracking system (Sensomotoric Instruments, Berlin). The stimulus was delivered using PRESENTATION (Neurobehavioral System) on a 17″ cultured monitor, with 1024 × 768 pixel resolution while the participants comfortably seated in chair at 75 cm away from it. Eye movement data were collected with sampling rate of 1250 Hz. The eye tracker recorded XY coordinates of eye gaze with an accuracy of 0.01°.

**Figure 1:**
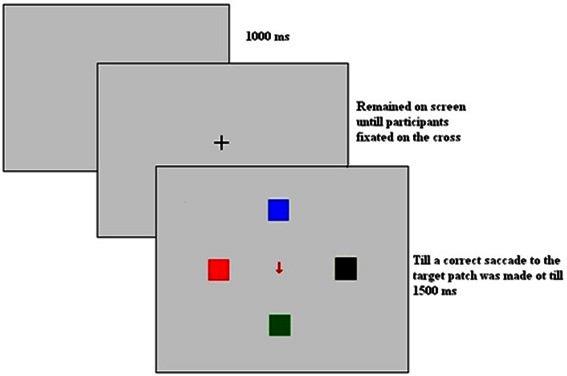
**A sample trial sequence for an incongruent trial**.

The experiment began with automatic calibration by presentation of a cross at 13 different locations on the screen. After successful calibration a fixation cross was presented which remained on the screen and it remained on the screen until participants fixated it. It was followed by a display consisting of four cultured squares and central arrow. Participants were instructed to look at the square matching the color of the arrow by making speeded eye movement toward it ignoring the square in which arrow was pointing. The display was made gaze contingent such that the display remained till the participants made a saccade to the correct square. However, the maximum time duration of the display was 1500 ms and in case participants didn't respond within this time a tone was presented to them and the trial ended. It was followed by blank screen for 1000 ms (see Figure 1).

### Data analysis

Eye tracking data were analysed using the BeGaze analysis software (Sensomotoric Instruments, Berlin). A saccade was defined as a movement of the eye more than 30°/s, following a velocity criterion from its present position in any direction. Each color patch was considered as an area of interest (AOI) for calculation of saccades and their latencies. Saccadic latency was calculated only for the correct trials. We did not consider those trials where the first saccade had landed on a wrong color patch. Fixations were counted if they fell on the color patch or very near it. This area was 135 × 135 in pixels. Each color patch was of 63 × 63 pixels. We also calculated saccadic error rates.

## Results

### Saccade latency

Saccadic latency or saccadic reaction time is the time lag between the onset of the display and the initiation of a saccade toward the correct color patch, i.e., the color patch matching the ink color of the central arrow. Data trimming involved exclusion of all the saccade latency less than 80 ms (anticipatory) and more than 1000 ms followed by further exclusion of saccade latencies which were more than two standard deviations from the final analysis.

**Figure 2:**
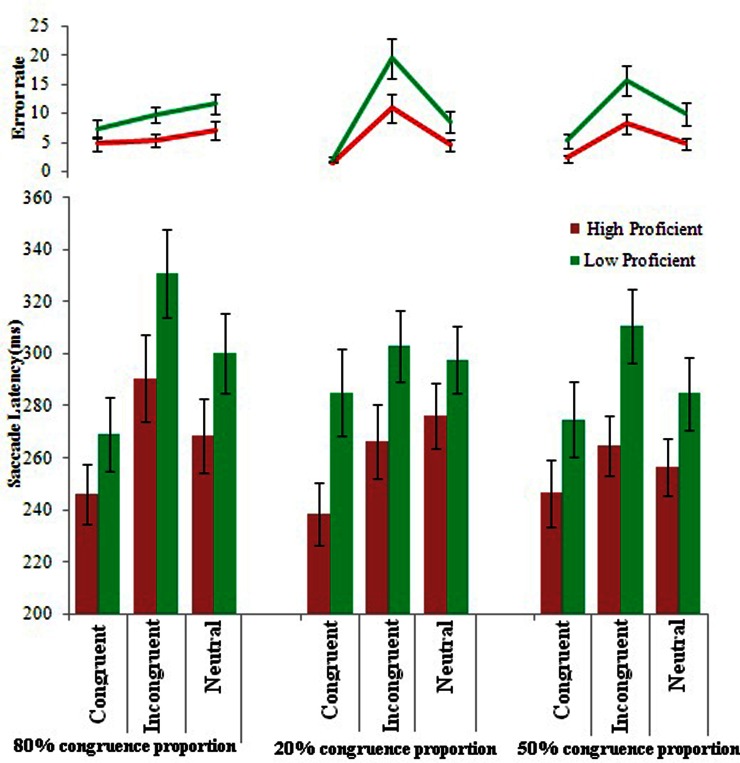
**Mean saccade latency (ms) and error rates for high and low proficient bilinguals for the saccadic arrow Stroop task in different congruence proportion conditions**.

A repeated measure of analysis of variance with congruency (congruent, incongruent, neutral) and congruence proportion (80% congruence, 20% congruent, and 50% congruence) as within subject factors and language proficiency(high and low proficiency bilinguals) as between subject factor was conducted on the saccade latency data. The high-proficient bilinguals were 33.6 ms faster in initiating a correct saccade than the low proficient bilinguals, *F*_(1, 54)_ = 5.0, *MSE* = 28396.406, *p* = 0.02, η^2^_*p*_ = 0.085 in general for all monitoring conditions. The main effect of congruency was significant, *F*_(2, 108)_ = 28.65, *MSE* = 2510.69, *p* = 0.001, η^2^_*p*_ = 0.347, revealing significantly higher saccade latency for the incongruent trials (294.3 ms) than the neutral (280.6 ms) and congruent(259.9 ms) trials respectively. The main effect of congruence proportions was not found significant, *F*_(2, 108)_ = 0.79, *MSE* = 6693.94, *p* = 0.45, η^2^_*p*_ = 0.04. The interaction between congruence proportion and language proficiency was not significant, *F*_(2, 108)_ = 0.018, *MSE* = 6693.94, *p* = 0.98, η^2^_*p*_ = 0.00. There was a significant interaction between congruence proportion and congruency, *F*_(4, 216)_ = 5.6, *MSE* = 1100.67, *p* = 0.001, η^2^_*p*_ = 0.095 (Figure 2). The interactions revealed significantly higher saccade latency for incongruent trials in 80 and 50% congruence proportion conditions than congruent trials in all the congruence proportion. The interaction between congruency and language proficiency was not significant, *F*_(2, 108)_ = 1.15, *MSE* = 172.185, *p* = 0.31, η^2^_*p*_ = 0.02. The three way interaction between language proficiency × congruency × proportion congruence was also not found to be significant, *F*_(4, 216)_ = 1.43, *MSE* = 931.0, *p* = 0.22, η^2^_*p*_ = 0.02 (see Table 3).

**Table 3:** **Mean saccadic latencies to the correct target, error rate, SIE (Stroop interference effect), and SFE (Stroop facilitation effect) for high and low proficient bilinguals (HPB and LPB) for the all the three monitoring conditions**.

	**Saccade latency (ms)**		**Error rate**	
	**HPB**	**LPB**	**HPB**	**LPB**
**80% CONGRUENT**				
Congruent	224.5 (60.8)	269.1 (72.5)	4.7 (6.0)	7.3 (7.9)
Incongruent	290.6 (87.2)	330.8 (90.4)	5.3 (5.6)	9.7 (7.0)
Neutral	268.6 (75.7)	300.2 (80.7)	7.0 (8.2)	11.6 (9.2)
SIE	21.9 (40.3)	30.5 (40.2)		
SFE	22.7 (49.7)	31.0 (46.4)		
**20% CONGRUENT**				
Congruent	238.3 (63.2)	285.0 (88.8)	1.3 (2.0)	2.0 (3.0)
Incongruent	266.3 (75.0)	302.9 (71.7)	10.8 (12.8)	19.9 (18.8)
Neutral	276.0 (67.5)	297.6 (67.3)	4.3 (5.1)	8.7 (9.4)
SIE	−9.7 (24.5)	5.3 (61.0)		
SFE^*^	37.6 (36.2)	12.6 (54.2)		
**50% CONGRUENT**				
Congruent	246.4 (68.2)	274.7 (75.9)	2.2 (3.4)	5.3 (6.3)
Incongruent	264.6 (61.0)	310.7 (76.2)	8.1 (8.5)	15.5 (13.7)
Neutral	256.5 (58.4)	284.7 (74.0)	4.7 (5.3)	9.9 (10.2)
SIE^*^	8.0 (22.0)	26.0 (37.0)		
SFE	10.1 (49.4)	10.0 (46.8)		

Standard deviations are given in parentheses.

* p < 0.05;

^**^p < 0.01.

To see further how the two groups differed in their conflict resolution ability we calculated Stroop interference effect (SIE) by subtracting saccadic latency on the neutral trials from the incongruent trials for each congruence proportion condition. The *t*-tests revealed that a significant difference between the SIE between the two groups was found only for 50% congruence proportion, *t*_(54)_ = −17.9, *p* = 0.03, *d* = 0.58 revealing that SIE was 18 ms smaller for the high-proficient bilinguals than the low proficient bilinguals. No significant difference in SIE was found for 80% congruence, *t*_(54)_ = −0.79, *p* = 0.42, *d* = 0.23 and 20% congruence proportion *t*_(54)_ = −1.2, *p* = 0.23, *d* = 0.32.

Likewise, the Stroop facilitation effect (SFE) was calculated (by subtracting saccade latency on the congruent trials from the neutral trials) for the two groups. The *t*-test revealed that high-proficient group showed significantly higher facilitation (37.6 ms) than low proficient bilinguals (12.6 ms) in the 20% congruent version, *t*_(54)_ = 2.0, *p* = 0.04, *d* = 0.53. However, there was no significant difference in SFE for the two groups in 80%, *t*_(54)_ = –0.64, *p* = 0.52, *d* = 0.17, and 50% congruence propotion, *t*_(54)_ = 0.006, *p* = 0.99, *d* = 0.00 (see Table 3).

### Error analysis

Any saccade toward any non-target square patches was counted as an error. A repeated measure of variance was conducted on the error with congruency (congruent, incongruent, neutral) and congruence proportion (80% congruent, 20% congruent, and 50% congruent) as within subject factors and language proficiency (high and low proficiency bilinguals) as a between subject factor. Low proficient bilinguals committed more errors than the high-proficient bilinguals, *F*_(1, 54)_ = 6.9, *MSE* = 379.4, *p* = 0.01, η^2^_*p*_ = 0.115. The main effect of congruency was significant, *F*_(2, 108)_ = 42.0, *MSE* = 93.33, *p* = 0.001, η^2^_*p*_ = 0.438, showing significantly higher errors on incongruent trials (11.6) than on congruent (3.8) and neutral trial(7.75). The main effect of congruence proportion was not found to be significant, *F*_(2, 108)_ = 0.058, *MSE* = 56.13, *p* = 0.94, η^2^_*p*_ = 0.01.

The interaction between congruency and language proficiency was found to be significant, *F*_(2, 108)_ = 4.0, *MSE* = 60.40, *p* = 0.021, η^2^_*p*_ = 0.06, revealing significantly higher error rates for low proficient bilinguals on the incongruent trials than high-proficient bilinguals on congruent and neutral trials. There was also a significant interaction between congruence proportion and congruency, *F*_(4, 216)_ = 21.6, *MSE* = 57.5, *p* = 0.001, η^2^_*p*_ = 0.28, showing significantly higher error rates for incongruent trials in the 20 and 50% congruence proportion than congruent trails in all three congruence proportion and neutral trials in 20% and 80% congruence proportion (Figure 2). Three way interaction between congruent proportion, congruency and language proficiency was not found to be significant, *F*_(4, 216)_ = 1.53, *MSE* = 28.64, *p* = 0.23, η^2^_*p*_ = 0.026.

## Discussion

In this study we tested if bilingual language fluency modulates monitoring in a modified oculomotor version of the Stroop task. Our main aim was to extend the predictions of the Bilingual Executive Advantage proposal with proficiency as a variable. We also wanted to replicate the monitoring account proposed by Costa et al. (2009) to test the claim that one can observe bilingual advantage only in scenarios where there is higher uncertainty. The participants were required to respond by making saccades toward the color patch that matched the color of the central arrow ignoring the square to which arrow was pointing. We obtained two important patterns of results. High-proficient bilinguals were overall faster on all types of trials in all monitoring blocks in general and there was a specific conflict advantage in the high monitoring condition. This pattern of results support Hilchey and Klein's proposal (Hilchey and Klein, 2011) showing an enhancement of the bilingual executive control abilities. This pattern of results successfully replicates the (Costa et al., 2009) findings in the oculomotor domain and with a novel task. Further, high-proficient bilinguals also committed less errors suggesting better oculomotor control. These results thus suggest superior cognitive control in bilinguals in many different domains and response systems and language proficiency modulates executive control in bilinguals. Our finding of a conflict advantage, suggests that this advantage emerges when attentional demand is higher (Bialystok and Martin, 2004; Hernández et al., 2010; Coderre et al., 2012; Singh and Mishra, 2012; Tse and Altarriba, 2012). Our results clearly show that high-proficient bilinguals were efficient in resisting capture of attention by the central cue toward the irrelevant square and directing their attention toward the target patch resolving any conflict. We argue that highly fluent bilinguals can modulate their selective attention in different monitoring contexts and show better interference control. The overall pattern of the results also are in harmony with studies that suggest better top-down attention control and goal maintenance in bilinguals (Colzato et al., 2008; Luk et al., 2010).

The highly fluent bilinguals were faster on all blocks of trials. However, Costa et al. (2009) had found a specific global RT advantage only in the block where congruent and incongruent trials were in equal numbers. Further, we observed a reduced conflict cost for the highly fluent bilinguals in this block, whereas Costa et al. (2009) had found such an effect only in one block that had 75% congruent trials. This discrepancy could be because of many reasons. First, we compared two different types of bilinguals with different proficiencies and Costa et al. (2009) had compared bilinguals with monolinguals. Secondly, we used a task that was a non-linguistic Stroop task whereas for Costa et al. (2009) it was a Flankers task with additional spatial cuing. Our blocks also had neutral trials. Costa et al. (2009) had found global RT advantage with 50% congruent trials. Importantly we measured oculomotor responses whereas for them it was a manual response. However, even with these important differences our results show overall speed advantage and supports Hilchey and Klein's theory of executive processing advantage (Hilchey and Klein, 2011). Further, studies should explore how different percentages of congruency affect this global RT advantages and conflict effects for different tasks.

Several previous studies with congruency proportion manipulations with the Stroop task have suggested that Stroop effect is generally larger for blocks where the proportion of congruent trials is higher (Logan and Zbrodoff, 1979; Lowe and Mitterer, 1982; Gratton et al., 1992; West and Baylis, 1998; see also Schmidt and Besner, 2008). That is because with large number of congruent trials compared to incongruent trials, participants develop a stimulus–response compatibility strategy and since very often the preceding trial of an in congruent trials happens to be congruent, they immediately cannot bring in additional control. In a mostly congruent block participants are also faster on congruent trials. In our case, both groups had higher SIEs in the 80% congruency block and this effect was low for the 20% congruency block. However, when the congruency was brought down to 50%, the high proficiency bilinguals suffered reduced conflict whereas this magnitude remained so for the low proficient bilinguals. This indicates that language proficiency modulates contextual effects of Stroop task.

Higher fluency bilinguals seem to have better goal directed attention control mechanisms compared to low proficient bilinguals. Colzato et al. (2008) suggested that bilinguals need not exert any inhibitory control to resolve interference, but they are better in maintenance of goals and executing task relevant action. In this sense, bilinguals have a better top-down action control strategy (also see Hernández et al., 2012). Our results indicate that high proficiency bilinguals were better at programming saccades toward the task relevant color patch while controlling interference from the symbolic cue. Our results seem to suggest that higher attentional demand aids in superior conflict resolution in the presence of an overall speed advantage. One way to look at these results is to suggest that higher proficiency bilinguals maintained task goals and selected the correct saccadic plan and therefore did not have to exert any inhibitory control. Higher proficient bilinguals showed better facilitation with congruent trials in the condition that had 20% congruent trials and reduced conflict cost where there were 50% congruent trials. The low proficient bilinguals did not show any such effects. The facilitation effects are again an indication of the overall superior executive control system.

It is important to examine the results considering the specific demands of the task used. As argued in the introduction, central arrows have two functions (Ristic and Kingstone, 2012) in orienting attention. First, they can reflexively affect spatial attention and may lead to automatic activation of the oculomotor system. Additionally, we had manipulated the monitoring by mixing the trials of different types. Participants had to keep the goal constantly in mind while programming a correct saccade and had to inhibit the reflexive saccade arising from the direction of the cue. Our results therefore support recent suggestions that bilingualism enhances the top down control system and allows greater control over goal maintenance and selection of the most appropriate response (Hernández et al., 2012). This top down control in the face of conflict involves the executive control system. Bilinguals' engage this system when the task demands are higher and their speed advantage is seen on all trial types. Importantly we have demonstrated that such effects generalize to the ocular responses and are not restricted to only tasks that demand a manual response.

Our results support modulatory effects of L2 proficiency on the bilingual advantage. Our study is in line with Tse and Altarriba (2012) who showed that both L1 and L2 proficiencies contributes to the enhancement of conflict resolution and goal maintenance in bilinguals. Our results go well with the study by Coderre et al. (2012) and make their findings even more lucid that it is enhanced conflict resolution in the bilinguals that leads to smaller SIEs. Since in our study we used non-linguistic arrow Stroop task the difference in the magnitude of suffered interference between the two groups of bilinguals can only be attributed to efficient conflict resolution modulated by their L2 proficiency.

On the same lines, Green (2011) proposes that one must look at the “behavioral ecology” of the bilinguals, particularly their switching and non-switching behaviors. It makes sense to think that bilinguals who use two languages more often and switch more may have developed a different kind of executive control system than other bilinguals. Taken together, these studies that have studied late adult bilinguals on different cognitive control tasks provide novel insights into the mechanisms of cognitive control.

There could be a possible limitation to our study related to the cognitive differences between the groups. We did not administer any objective task to measure language fluency, and depended on the outcome of the self-administered language questionnaire. The subjectivity of this task could have been a confound in participant selection.

In conclusion then, we have shown that bilingual language proficiency can modulate oculomotor control in a conflict task, and this indicates their superior executive control ability. Future studies should explore how bilinguals with different language abilities and age of acquisitions of L2 develop different executive control system. In this scenario, the language environment of the bilinguals and the use of the two languages seem crucial for interpreting experimental effects obtained on different non-linguistic attention tasks.

### Conflict of interest statement

The authors declare that the research was conducted in the absence of any commercial or financial relationships that could be construed as a potential conflict of interest.

## References

[B1] Bialystok E. (2010). Global-local and trail-making tasks by monolingual and bilingual children: beyond inhibition. Dev. Psychol. 46, 93–105 10.1037/a0015466 20053009 PMC2805165

[B2] Bialystok E. Craik F. I. M. Grady C. Chau W. Ishii R. Gunji A. (2005). Effect of bilingualism on cognitive control in the Simon task: evidence from MEG. Neuroimage 24, 40–49 10.1016/j.neuroimage.2004.09.044 15588595

[B3] Bialystok E. Craik F. I. M. Ryan J. (2006). Executive control in a modified antisaccade task: effects of aging and bilingualism. J. Exp. Psychol. Learn Mem. Cogn. 32, 1341–1352 10.1037/0278-7393.32.6.1341 17087588

[B4] Bialystok E. Feng X. (2009). Language proficiency and executive control in proactive interference: evidence from monolingual and bilingual children and adults. Brain Lang. 109, 93–100 10.1016/j.bandl.2008.09.001 18834625 PMC2699211

[B5] Bialystok E. Martin M. M. (2004). Attention and inhibition in bilingual children: evidence from the dimensional change card sort task. Dev. Sci. 7, 325–339 10.1111/j.1467-7687.2004.00351.x 15595373

[B6] Bialystok E. Viswanathan M. (2009). Components of executive control with advantages for bilingual children in two cultures. Cognition 112, 494–500 10.1016/j.cognition.2009.06.014 19615674 PMC2755257

[B7] Blumenfeld H. Marian V. (2007). Constraints on parallel language activation in bilingual spoken language processing: examining proficiency and lexical status using eye-tracking. Lang. Cogn. Process. 22, 633–660 10.1080/0169096060100074619586268

[B8] Calabria M. Hernandez M. Branzi F. M. Costa A. (2011). Qualitative differences between bilingual language control and executive control: evidence from task-switching. Front. Psychol. 2:399 10.3389/fpsyg.2011.00399 22275905 PMC3257869

[B9] Carlson S. M. Meltzoff A. N. (2008). Bilingual experience and executive functioning in young children. Dev. Sci. 11, 282–298 10.1111/j.1467-7687.2008.00675.x 18333982 PMC3647884

[B10] Coderre E. Van Heuven W. J. B. Conklin K. (2012). The timing and magnitude of Stroop interference and facilitation in monolinguals and bilinguals. Biling. Lang. Cogn. 1, 22 10.1017/S1366728912000405 23483406 PMC3590568

[B11] Colzato L. S. Bajo M. T. van den Wildenberg W. Paolieri D. Nieuwenhuis S. La Heij W. (2008). How does bilingualism improve executive control? A comparison of active and reactive inhibition mechanisms. J. Exp. Psychol. Learn. Mem. Cogn. 34, 302–312 10.1037/0278-7393.34.2.302 18315407

[B12] Costa A. Hernández M. Costa-Faidella J. Sebastián-Gallés N. (2009). On the bilingual advantage in conflict processing: now you see it, now you don't. Cognition 113, 135–149 10.1016/j.cognition.2009.08.001 19729156

[B13] Costa A. Hernández M. Sebastián-Gallés N. (2008). Bilingualism aids conflict resolution: evidence from the ANT task. Cognition 106, 59–86 10.1016/j.cognition.2006.12.013 17275801

[B14] Driver J. Davies M. Ricciardelli P. (1999). Gaze perception triggers reflective visuo-spatial orienting. Vis. Cogn. 6, 509–540

[B15] Emmorey K. Luk G. Pyers J. E. Bialystok E. (2009). The source of enhanced cognitive control in bilinguals: evidence from bimodal bilinguals. Psychol. Sci. 19, 1201–1206 10.1111/j.1467-9280.2008.02224.x 19121123 PMC2677184

[B16] Festman J. Münte T. (2012). Cognitive control in Russian–German bilinguals. Front. Psychol. 3:115 10.3389/fpsyg.2012.00115 22529831 PMC3328798

[B17] Festman J. Rodriguez-Fornells A. Munte T. F. (2010). Individual differences in control of. language interference in late bilinguals are mainly related to general executive abilities. Behav. Brain Funct. 6:5 10.1186/1744-9081-6-5 20180956 PMC2830994

[B18] Friesen C. K. Kingstone A. (1998). The eyes have it! Reflexive orienting is triggered by nonpredictive gaze. Psychon. Bull. Rev. 5, 490–495

[B19] Friesen C. K. Ristic J. Kingstone A. (2004). Attentional effects of counterpredictive gaze and arrow cues. J. Exp. Psychol. Hum. Percept. Perform. 30, 319–329 10.1037/0096-1523.30.2.319 15053691

[B20] Gratton G. Coles M. G. Donchin E. (1992). Optimizing the use of information: strategic control of activation of responses. J. Exp. Psychol. Gen. 121, 480–506 10.1037/0096-3445.121.4.480 1431740

[B21] Green D. W. (1998). Mental control of the bilingual lexico-semantic system. Biling. Lang. Cogn. 1, 67–81 10.1017/S1366728998000133

[B22] Green D. W. (2011). Language control in different contexts: the behavioral ecology of bilingual speakers. Front. Psychol. 2:103 10.3389/fpsyg.2011.00103 21779260 PMC3132677

[B23] Guo T. Misra M. Tam J. W. Kroll J. F. (2012). On the time course of accessing meaning in a second language: an electrophysiological investigation of translation recognition. J. Exp. Psychol. Learn. Mem. Cogn. 38, 1165–1186 10.1037/a0028076 22686844 PMC3925769

[B24] Guo T. Peng D. (2006). ERP evidence for parallel activation of two languages in bilingual speech production. Neuroreport 17, 1757–1760 10.1097/01.wnr.0000246327.89308.a5 17164659

[B25] Hernández M. Costa A. Fuentes L. J. Vivas A. B. Sebastian-Galles N. (2010). The impact of bilingualism on the executive control and orienting networks of attention. Biling. Lang. Cogn. 13, 315–325 10.1017/S1366728909990010

[B26] Hernández M. Costa A. Humphreys G. W. (2012). Escaping capture: bilingualism modulates distraction from working memory. Cognition 122, 37–50 10.1016/j.cognition.2011.08.002 21890125

[B27] Hilchey M. D. Klein R. M. (2011). Are there bilingual advantages on nonlinguistic interference tasks? Implications for the plasticity of executive control processes. Psychon Bull. Rev. 18, 625–658 10.3758/s13423-011-0116-7 21674283

[B28] Hodgson T. L. Parris B. A. Gregory N. J. Jarvis T. (2009). The saccadic Stroop effect: evidence for involuntary programming of eye movements by linguistic cues. Vision Res. 49, 569–574 10.1016/j.visres.2009.01.001 19183561 PMC2724027

[B29] Jared D. Kroll J. F. (2001). Do bilinguals activate phonological representations in one or both of their languages when naming words? J. Mem. Lang. 44, 2–31

[B30] Kovács Á. M. Mehler J. (2009). Cognitive gains in 7-montholdbilingual infants. Proc. Natl. Acad. Sci. U.S.A. 106, 6556–6560 10.1073/pnas.0811323106 19365071 PMC2672482

[B31] Kunde W. Skirde S. Weigelt M. (2011). Trust my face: cognitive factors of head fakes in Sports. J. Exp. Psychol. Appl. 17, 110–127 10.1037/a0023756 21604910

[B32] Langton S. R. H. Bruce V. (1999). Reflexive visual orienting in responseto the social attention of others. Vis. Cogn. 6, 541–568

[B33] Langton S. R. Watt R. J. Bruce I. I. (2000). Do the eyes have it? Cues to the direction of social attention. Trends Cogn. Sci. 4, 50–59 10.1016/S1364-6613(99)01436-9 10652522

[B34] Logan G. D. Zbrodoff N. J. (1979). When it helps to be misled: facilitative effects of increasing the frequency of conflicting stimuli in a Stroop-like task. Mem. Cogn. 7, 166–174 10.3758/BF03197535

[B35] Lowe D. G. Mitterer J. O. (1982). Selective and divided Attention in a Stroop task. Can. J. Psychol. 36**,** 684–700. 7159848 10.1037/h0080661

[B36] Luk G. Anderson J. A. E. Craik F. I. M. Grady C. Bialystok E. (2010). Distinct neural correlates for two types of inhibition in bilinguals: response inhibition versus interference suppression. Brain Cogn. 74, 347–357 10.1016/j.bandc.2010.09.004 20965635

[B37] Luk G. De Sa E. Bialystok E. (2011). Is there a relation between onset age of bilingualism and enhancement of cognitive control? Biling. Lang. Cogn. 14, 110–127

[B38] Martin-Rhee M. M. Bialystok E. (2008). The development of two types of inhibitory control in monolingual and bilingual children. Biling. Lang. Cogn. 11, 81–93 10.1017/S1366728907003227

[B39] Mishra R. K. Hilchey M. D. Singh N. Klien R. M. (2012). On the time course of exogenous cueing effects in bilinguals: higher proficiency in a second language is associated with more rapid endogenous disengagement. Q. J. Exp. Psychol. 65, 1502–1510 10.1080/17470218.2012.657656 22512692

[B40] Posner M. I. (1980). Orienting of attention. J. Exp. Psychol. 32, 3–25 10.1080/00335558008248231 7367577

[B41] Posner M. I. (1994). Attention: the mechanisms of consciousness. Proc. Natl. Acad. Sci. U.S.A. 91, 7398–7403 8052596 10.1073/pnas.91.16.7398 PMC44408

[B42] Ristic J. Friesen C. K. Kingstone A. (2002). Are eyes special? It depends on how youlook at it. Psychon. Bull. Rev. 9, 507–513 10.3758/BF03196306 12412890

[B43] Ristic J. Kingstone A. (2012). A new form of human spatial attention: automated symbolic orienting. Vis. Cogn. 20, 244–264 10.1080/13506285.2012.658101

[B44] Schmidt J. R. Besner D. (2008). The Stroop effect: why proportion congruent has nothing to do with congruency and everything to do with contingency. J. Exp. Psychol. Learn Mem. Cogn. 34**,** 514–523 10.1037/0278-7393.34.3.514 18444752

[B45] Singh N. Mishra R. K. (2012). Does language proficiency modulate oculomotor control? Evidence from Hindi–English bilinguals. Biling. Lang. Cogn. 15, 771–781

[B46] Soveri A. Rodriguez-Fornells A. Laine M. (2011). Is there a relationship between language switching and executive functions in bilingualism? Introducing a within-group analysis approach. Front. Psychol. 2:183 10.3389/fpsyg.2011.00183 21869878 PMC3150725

[B47] Sunderman G. Priya K. (2012). Translation recognition in highly proficient Hindi-English bilinguals. The influence of different scripts but connectable phonologies. Lang. Cogn. Process. 27, 1265–1285 10.1080/01690965.2011.596420

[B48] Tao L. Marzecová A. Taft M. Asanowicz D. Wodniecka Z. (2011). The efficiency of attentional networks in early and late bilinguals: the role of age of acquisition. Front. Psychol. 2, 1–19 10.3389/fpsyg.2011.00123 21713011 PMC3114252

[B49] Thierry G. Wu Y. J. (2010). Chinese–english bilinguals reading english hear chinese. J. Neurosci. 30, 7646–7651 10.1523/JNEUROSCI.1602-10.2010 20519539 PMC6632379

[B50] Tipples J. (2002). Eye gaze is not unique: automatic orienting in response to uninformative arrows. Psychon. Bull. Rev. 9, 314–318 10.3758/BF03196287 12120794

[B51] Tse C.-S. Altarriba J. (2012). The effects of first- and second-language proficiency on conflict resolution and goal maintenance in bilinguals: evidence from reaction time distributional analyses in a Stroop task. Biling. Lang. Cogn. 15, 663–676 10.1017/S1366728912000077

[B52] Van Hell J. Dijkstra A. (2002). Foreign language knowledge can influence native language performance. Psychon. Bull. Rev. 9, 780–789 10.3758/BF03196335 12613683

[B53] West R. Baylis G. C. (1998). Effects of increased response dominance and contextual disintegration on the Stroop interference effect in older adults. Psychol. Aging. 13, 206–217 10.1037/0882-7974.13.2.206 9640582

[B54] Ye Z. Zhou X. (2009). Executive control in language processing. Neurosci. Biobehav. Rev. 33, 1168–1177 10.1016/j.neubiorev.2009.03.003 19747595

